# Adaptation of Anaerobic Digestion Microbial Communities to High Ammonium Levels: Insights from Strain-Resolved Metagenomics

**DOI:** 10.1021/acs.est.3c07737

**Published:** 2023-12-19

**Authors:** Luca Bucci, Gabriele Ghiotto, Guido Zampieri, Roberto Raga, Lorenzo Favaro, Laura Treu, Stefano Campanaro

**Affiliations:** ‡ Department of Biology (DIBIO), University of Padova, Via Ugo Bassi 58/B, 35131 Padova, Italy; § Department of Civil, Environmental and Architectural Engineering (ICEA), University of Padova, Via Marzolo 9, 35131 Padova, Italy; ∥ Department of Agronomy Food Natural Resources Animals and Environment (DAFNAE), University of Padova, Campus Agripolis, Viale dell’Università 16, 35020 Legnaro, Italy

**Keywords:** anaerobic digestion, ammonia inhibition, stress adaptation, metagenomics, single nucleotide variants

## Abstract

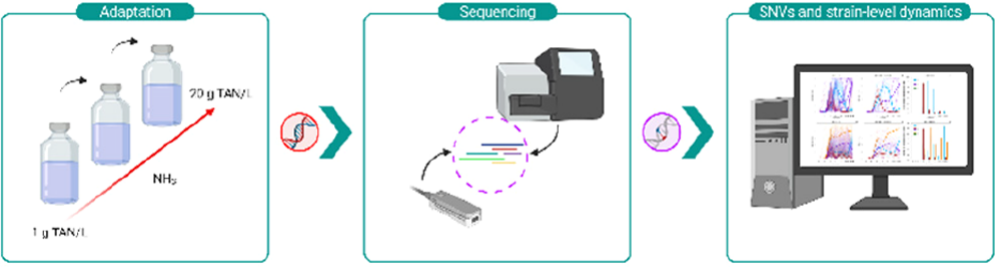

Ammonia release from proteinaceous feedstocks represents the main inhibitor of the anaerobic digestion (AD) process, which can result in a decreased biomethane yield or even complete failure of the process. The present study focused on the adaptation of mesophilic AD communities to a stepwise increase in the concentration of ammonium chloride in synthetic medium with casein used as the carbon source. An adaptation process occurring over more than 20 months allowed batch reactors to reach up to 20 g of NH_4_^+^ N/L without collapsing in acidification nor ceasing methane production. To decipher the microbial dynamics occurring during the adaptation and determine the genes mostly exposed to selective pressure, a combination of biochemical and metagenomics analyses was performed, reconstructing the strains of key species and tracking them over time. Subsequently, the adaptive metabolic mechanisms were delineated by following the single nucleotide variants (SNVs) characterizing the strains and prioritizing the associated genes according to their function. An in-depth exploration of the archaeon *Methanoculleus bourgensis* vb3066 and the putative syntrophic acetate-oxidizing bacteria *Acetomicrobium* sp. ma133 identified positively selected SNVs on genes involved in stress adaptation. The intraspecies diversity with multiple coexisting strains in a temporal succession pattern allows us to detect the presence of an additional level of diversity within the microbial community beyond the species level.

## Abstract 2

This study explores the biological mechanisms behind ammonia inhibition in anaerobic digestion systems, achieving the highest level of resistance reported in microbial communities and unveiling insights into the molecular adaptation strategies against inhibitors.

## 1 Introduction

Anaerobic digestion (AD) is mediated by a complex microbial community responsible for the conversion of organic carbon to compounds CO_2_ and CH_4_. Anaerobic archaea mediate the final step, starting from acetate, H_2_ and CO_2_, or methylated C1 compounds in the acetoclastic, hydrogenotrophic, and methylotrophic methanogenesis, respectively.^1−5^ The process efficiency can be affected by several parameters, like the temperature, pH, and O_2_, salt, and ammonia concentrations.^3,6,7^ The main source of instability in AD is ammonia inhibition.^8^ The processing of N-rich organic wastes (e.g., waste-activated sludge, manure, and food-processing plants) can result in high ammonia release, which may lead to an unbalanced digestion process.^9^

As a result of the chemical equilibrium between ammonia (NH_3_) and ammonium (NH_4_^+^), governed by the temperature and pH, these two forms do not exert equal levels of toxicity on the microbiome. Total ammonia nitrogen (TAN, NH_3_ + NH_4_^+^) and free ammonia nitrogen (FAN, NH_3_) are used to quantify ammonia, with FAN being recognized as the most toxic form.^10,11^ Generally, as the pH decreases, ammonia stress is partially alleviated, leading to a reduction in the FAN concentration and driving the process toward a state known as the “inhibited steady state”.^1,12−14^ Microorganisms that bear the brunt of ammonia stress, notably acetoclastic archaea,^15^ may undergo acetate metabolism sweep at elevated ammonia concentrations. This shift can promote a dynamic transition from acetoclastic methanogenesis to syntrophic acetate oxidation (SAO), resulting in the production of H_2_ and CO_2_.^13,15^ Specifically, SAO occurs via the reverse Wood–Ljungdahl (WL) pathway, a biochemical route studied in *Schnuerera ultunensis* or *Syntrophaceticus schinkii*,^16^ and/or through the alternative glycine synthase–reductase (GSR) pathway.^17,18^

Ammonia toxicity is a general mechanism responsible for unbalancing intracellular pH and determining high-energy requirements. In particular, FAN diffuses into cells, absorbs intracellular protons, and partially converts to NH_4_^+^. Cells respond to the consequent pH increase by enhancing the activity of cation antiporters and internalizing H^+^, despite essential cations. This subtracts more energy from the metabolism, thus potentially inhibiting specific reactions, like methanogenesis.^10,19^ If intracellular NH_4_^+^ is particularly high, its accumulation destabilizes intracellular pH and disturbs the dynamics of other cations (such as Mg^+^), causing cytotoxicity. Specifically, methanogens exhibit a heightened vulnerability to ammonia toxicity, owing to the absence of peptidoglycan in their cell wall and the oxidation of critical cofactors integral to the methanogenic process.^12^ When microorganisms are exposed to toxic ammonia concentrations, random mutations conferring an advantage to survival can be positively selected. These adaptive mutations empower microbes to enhance their toxicity tolerance^20^ by modulating the structure and/or activity of proteins involved in the response to the stressor. In this context, exposing microorganisms to escalating concentrations of ammonia can decrease the degree of ammonia inhibition.^8^

The process of microbial exposure up to 20 g of NH_4_^+^ N/L has not been previously employed. To our knowledge, genomic heterogeneity existing within the AD microbiome received very little attention in previous studies, and the role of variants is still unexplored. The sole precedent exists in a study investigating the response of gut microbial communities to antibiotic perturbations, where phased variants were linked to distinct strains belonging to the same species.^21^ The longitudinal monitoring of single nucleotide variant (SNV) trajectories over time facilitated a comparative analysis of genetic dynamics and ecological fluctuations at the species level after the introduction of stressors. Therefore, it is extremely relevant to consider microbial dynamics at both species and strain levels, with a particular focus on emerging mutations. In the present study, the evolution of the microbial behavior in response to a stepwise growing concentration of ammonia was investigated. The purpose was to examine the extent to which the microbial community could withstand stress in a context of genomic adaptation for obtaining a deep understanding of ammonia-tolerant methanogenic consortia. To understand how the microbial community composition changed, strain-resolved metagenomics was applied. For the first time, the variant calling incorporated shotgun metagenomic reads to trace strains by identifying distinctive patterns of alleles across SNVs within a species.^22,23^ Furthermore, the strain deconvolution strategy extracts strain genotypes from shotgun metagenomic data based on allele frequencies.^24,25^ This combination unveiled fine-scale evolutionary mechanisms, functional dynamics, and metabolic variations that could contribute to the selection of resistant microorganisms.

## 2 Materials and Methods

### 2.1 Inoculum and Culture Maintenance

The starting inocula were collected from a mesophilic full-scale AD plant (section S1 of the Supporting Information) characterized by a high ammonia content (>4 g of NH_4_^+^ N/L). Two tanks of the in-series reactor, fermentation (F) and post-fermentation (P), were sampled, respectively, operated at 42.3 and 41.2 °C. After sampling, the starting point of the adaptation procedure was defined. Inocula were kept in the dark at 42 ± 1 °C and fed weekly with nearly 1 g/L nutrient broth (NB, Merck Millipore, Billerica, MA, U.S.A.) for 7 weeks before their inoculations. Phylum-level taxonomic classification (related to the day of experimental setup) of the inocula is reported in Figure S1 of the Supporting Information.

Anaerobic cultures were grown in the basal anaerobic medium (BAm), prepared as described by Angelidaki et al.^26^ The used carbon sources are described in the next section. Before the inoculation, sodium sulfide nonahydrate (Na_2_S·9H_2_O, Acros Organics, Belgium) solution was added as a reducing agent, together with a double volume of vitamin solution.

### 2.2 Adaptation Procedure

To maintain anaerobic conditions, inocula were seeded inside a glovebox (MBRAUN MB200, Germany) with a N_2_ atmosphere. After sieving to remove large particles, inocula were diluted with BAm at 20:80 (v/v) inside 118 mL serum glass bottles with 40 mL working volume and incubated in the dark at 42 ± 1 °C. Subsequent re-inocula every 21 days were defined as cultivation generations. Each generation was re-inoculated with stepwise increasing concentrations of ammonia, in the form of NH_4_Cl (Sigma-Aldrich, St. Louis, MO, U.S.A.). Starting from the second generation, casein (from bovine milk, Sigma-Aldrich) was used as the main carbon source (Table S1 of the Supporting Information) in the F/P2 reactors. Glucose (Carlo Erba Reagents, Italy) and acetate (acetic acid glacial, Carlo Erba Reagents) were also tested (Table S1 of the Supporting Information). Yeast extract (YE, 26 Oxoid Limited, London, U.K.) and vitamin solution were used to sustain microbial growth. In the re-inoculation process, biogas production and pH were measured and samples were collected and immediately frozen at −20 °C. Starting from the 10th generation, casein-fed cultures were re-inoculated at two different NH_4_Cl concentrations, with 2 g/L difference among them (Table S2 of the Supporting Information), and grown independently. The adaptation reached the 27th generation, corresponding to around 19.8 and 20.3 calculated g of NH_4_^+^ N/L.

### 2.3 Physicochemical Analyses

Gas measurements were initially conducted every 15 days and then weekly starting from the ninth generation. The volume of the biogas overpressure generated during the fermentation was measured using a graduated syringe. Gas and volatile fatty acid (VFA) compositions were determined using a gas chromatograph (8860 GC, Agilent Technologies, Santa Clara, CA, U.S.A.), as extensively described in the Supporting Information.

Biochemical parameters, including pH, TAN, and total Kjeldahl nitrogen (TKN), were determined according to American Public Health Association (APHA) standard methods for the examination of water and wastewater^27^ up to the 25th generation of the casein-fed reactors. TKN was measured via the macro-Kjeldahl method. Chloride ion (Cl^–^) content across generations was estimated on the basis of salt supplementation levels (Table S2 of the Supporting Information). FAN was calculated according to the following equation:^28^
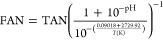
1

### 2.4 DNA Extraction and Sequencing

DNA extractions from pellets were performed in duplicates using the DNeasy PowerSoil Pro Kit (Qiagen, Germany) following the instructions of the manufacturer. After the extraction, the resulting DNA quality was evaluated using a NanoDrop ND-1000 ultraviolet–visible (UV–vis) spectrophotometer (Thermo Fisher Scientific, Waltham, MA, U.S.A.) and quantified with the Qubit fluorometer using the Qubit dsDNA high sensitivity assay kit (Invitrogen, Waltham, MA, U.S.A.). Libraries were prepared by adopting the Nextera DNA Flex Library Prep Kit (Illumina, Inc., San Diego, CA, U.S.A.) and the SQK-RBK004 rapid barcoding kit (Oxford Nanopore Technologies, U.K.). Sequencing was then performed with both Illumina NovaSeq 6000 platform (2 × 150 bp, paired end) and Oxford Nanopore MinION (section S3 of the Supporting Information) at the Next-Generation Sequencing (NGS) Facility of the Department of Biology (University of Padua, Italy).

Sequencing and bioinformatics analyses were carried out on time points chosen as representative of the adaptation process based on biochemical data (sheets S1–S3 of the Supporting Information). Apart from the selected time points, the adaptation process of the ammonia increase has been further continued.

### 2.5 Metagenomic Data Analysis

Bioinformatics analysis of NGS data was performed using a genome-centric metagenomics approach. Illumina reads were filtered with Trimmomatic v0.39^29^ and merged with BBMerge.^30^ Short reads were co-assembled with Megahit version 1.2.9,^31^ while long reads were co-assembled with Flye version 2.9-b1768.^32^ Pilon version 1.23^33^ was employed for assembly correction, and Quickmerge version 0.3^34^ was employed for generating the hybrid Nanopore–Illumina assembly. The binning process was performed using Concoct version 1.1.0,^35^ MetaBAT version 2.15,^36^ MetaBAT2 version 2.15,^37^ and VAMB version 3.0.2-1.^38^ For coverage calculations, bowtie2 version 2.4.5^39^ and SAMtools version 1.14^40^ were employed. Retrieved bins were dereplicated with dRep version 3.4.0.^41^ Quality and relative abundance (RA) of resulting metagenome-assembled genomes (MAGs) were accessed with CheckM version 1.2.2.^42^ Taxonomic classification was obtained with GTDB-Tk version 2.1.1.^43^ Open reading frames (ORFs) were predicted with Prodigal version 2.6.3,^44^ followed by functional annotation with eggNOG-mapper version 2.1.2.^45^ Kyoto Encyclopedia of Genes and Genomes (KEGG)^46^ and MicrobeAnnotator version 2.0.4^47^ were used to assess the completeness of pathways. Phylogenetic analysis was performed with Phylophan version 3.0,^48^ and the resulting Newnick tree was visualized with iTOL (https://itol.embl.de/). More details are given in section S4 of the Supporting Information. Raw sequencing data were uploaded to the National Center for Biotechnology Information (NCBI) Sequence Read Archive database (PRJNA975341).

### 2.6 Variant- and Strain-Level Analyses

As a result of the failure of the glucose- and acetate-fed batches, only the casein-fed batches were considered for variant and strain analyses (see section 3.1). First, SNVs were identified using InStrain version 1.6.3^22^ (more details are presented in section S5 of the Supporting Information). A set of seven MAGs was selected by prioritizing genomes with high sequencing coverage and number of variants detected by InStrain.^22^ After the selection, variants were grouped into clusters based on their frequency over time, as described by Ghiotto et al.^50^ The Mann–Whitney *U* test was applied to assess the accumulation of non-synonymous single nucleotide variants (nsSNVs) in genes associated with the pathways of interest in a MAG with respect to the overall gene population in the same MAG. The Grantham distance^49^ was calculated for each SNV, reflecting physical and chemical differences between reference and mutated amino acids (sheet S10 of the Supporting Information). Variants with a distance of >70 were considered to have medium–high impact on the structure of the protein. Then, STRONG^24^ was applied individually to the seven selected MAGs, with default parameters, to infer the strains belonging to the respective species and their dynamics across samples.

## 3 Results and Discussion

To investigate the biological mechanisms associated with microbial inhibition and resistance to high ammonia levels, a batch reactor system was settled and exposed to a serial increment in the NH_4_Cl concentration. This approach induced a gradual response to the stressor and promoted a slow adaptation of the microbial community. Three substrates were selected to set up the initial batches (glucose, acetate, and casein), with the goal of testing how different carbon sources can influence the inoculum adaptation. The stochastic nature governing the selection process led to the independent evolution of the microorganisms in the reactors, thereby preventing the application of replicates. Thus, all of the conditions under investigation were considered independently. To monitor microbial adaptation, a suite of metagenomics-based approaches was implemented. Additionally, observations discussed in this work are based on variations in RA, without considering total cell counts. Therefore, a low CH_4_ productivity may be related to a decrease in the cell counts and viability.

### 3.1 Medium Selection According to the Reactor Performance

Starting from the original microbiota, the first re-inoculum in synthetic media supplemented with C sources (second generation) showed an overall reduction in CH_4_ production (sheet S1 of the Supporting Information). This drop could be linked with the lag phase after the change of medium composition. In fact, from the third generation, CH_4_ production increased. As a result of the fluctuating behavior, generation three can be considered the real starting point for observing the impact of ammonia on the CH_4_ yield. A gradual decrease in methane production was observed in the following generations, coherent with the stepwise increase in TAN (sheet S2 of the Supporting Information). This reduction reflects the inhibitory effect of high ammonium.^51^ Responses of glucose- and acetate-fed reactors during the experiment suggested failures of both systems (section S6 of the Supporting Information). The ammonia level in the first generation was lowered in comparison to the TAN in the inoculum (reported as generation zero), to mitigate the stress induced on the community while shifting from the feedstock in the reactor to the growth medium. Moreover, the TAN increase at each step was finely tuned to limit the stress and to give the microbiome the possibility to adapt through a simplified adaptive laboratory evolution (ALE) process.^52^ In summary, the gradual adaptation strategy adopted strongly contributed to the arising mutations and their contribution to the methanogenic consortia survival.

### 3.2 Slow Adaptation Favored Microbial Tolerance to High TAN

**Figure 1:**
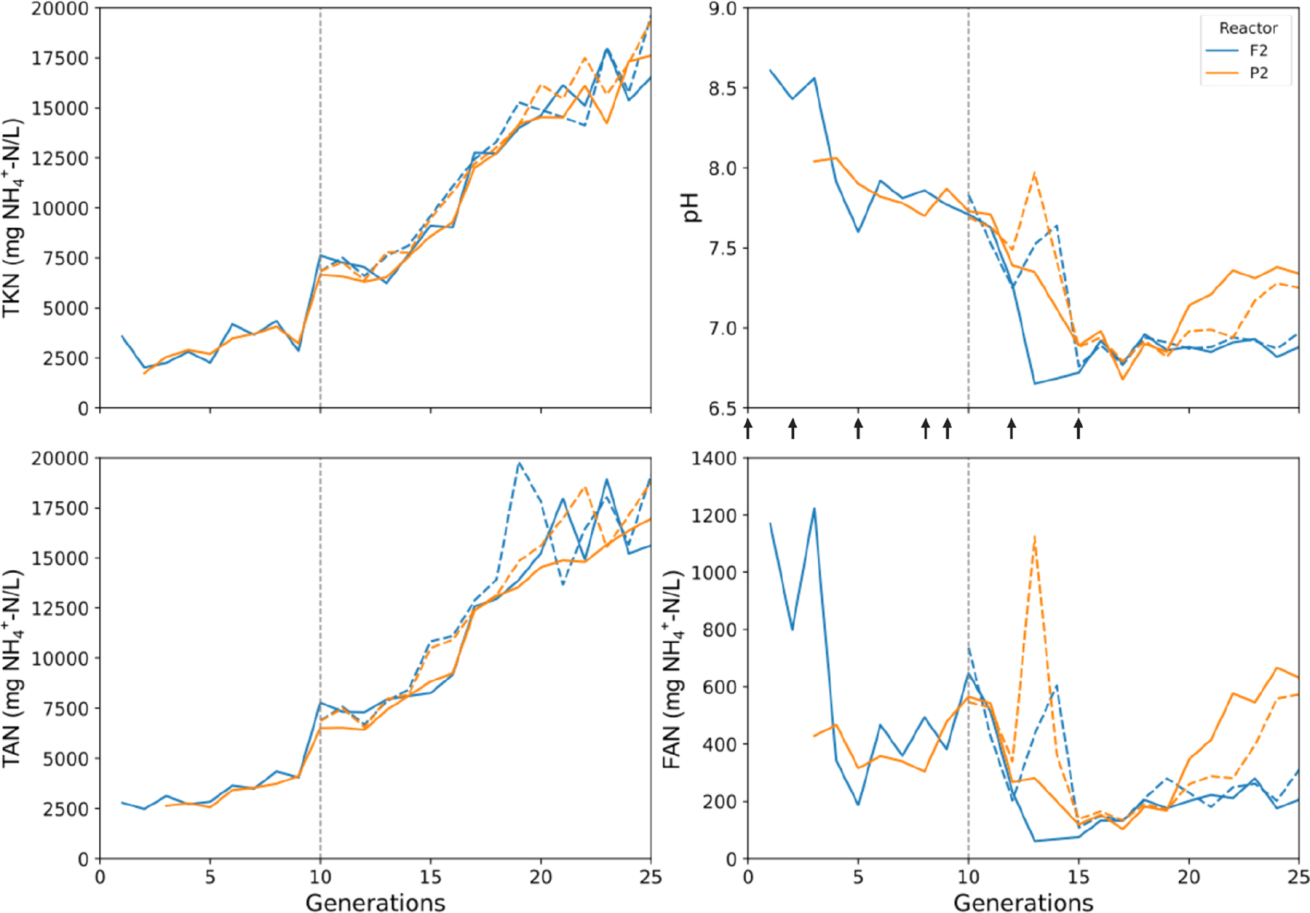
Most relevant biochemical parameters, TKN, pH, TAN, and FAN, were measured for each generation. Arrows mark the sampled generations for metagenomics analysis. The two casein-fed reactors, F2 (fermentation inoculum) and P2 (post-fermentation inoculum), are highlighted by blue and orange colors, respectively. Starting from the 10th generation, batches were split in two. Dashed lines refer to the batches where the ammonia concentration was kept at 0.5 NH_4_^+^ N/L higher than the others in full lines, and vertical dashed lines indicate their starting point.

Biologically relevant parameters were monitored at the end of each generation (Figure 1 and sheets S1–S3 of the Supporting Information). As expected, increasing values of TKN and TAN were recorded. The pH underwent a slow and consistent decrease, stabilizing around 6.8 ± 0.07 (mean ± standard deviation), consequently affecting the FAN. Even though TAN increased to extremely high values, pH more effectively governed FAN progression, determining its limited increase (Figure 1). However, measured FAN is within the reported inhibitory range of microbial growth (up to 1450 mg of NH_4_^+^ N/L).^53^ Specifically, P2 reactors underwent a drop in cumulative methane production concurrently with the FAN increment (sheet S1 of the Supporting Information). Throughout the time course, high values of TAN were also reached (Figure 1), considering that up to 14 g of NH_4_^+^ N/L is reported in the literature as the inhibiting threshold.^54^ However, our results demonstrated that AD microbes can endure up to 20 g of NH_4_^+^ N/L if allowed to gradually adapt (Figure 1). High TAN levels when obtained with NH_4_Cl supplementation also result in osmotic stress. Proteins involved in potassium uptake and compatible solute production are under strong selective pressure, and as explored in section 3.4, this could play a crucial role in salt adaptation.

### 3.3 Metagenomic Results and Microbiome Specialization

**Figure 2:**
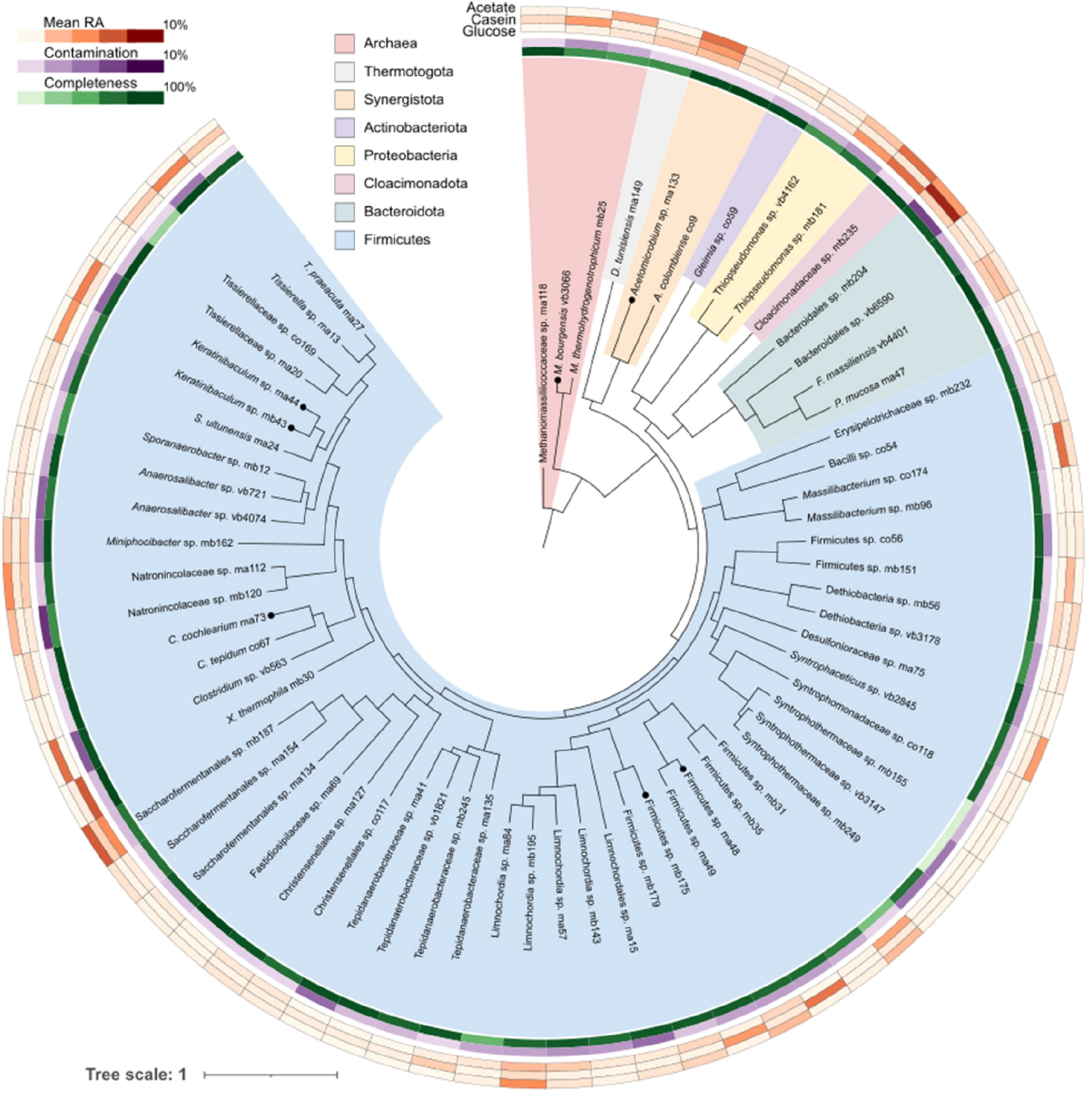
Overview of the microbial community composition having a 1% or higher RA in at least one generation. MAGs are represented according to their phylogenetic relationship. Completeness and contamination of the MAGs and average RA within different feedstocks are reported in the external circles. MAGs selected for SNV analysis that survived up to the highest ammonia concentration are marked with black dots in the phylogenetic tree.

A hybrid assembly of 618.24 Mb was obtained by combining long and short reads. Taxonomic assignment revealed a diverse microbiome with 179 MAGs, primarily consisting of 172 bacterial (96.09%) and 7 archaeal (3.91%) MAGs, dominated by Firmicutes (72.07%). *Methanoculleus bourgensis* vb3066 was the prominent methanogen species with a mean RA of 4.33% (Figure 2 and section S7 of the Supporting Information).

The high RA of *M. bourgensis* vb3066 recorded in the present study is probably favored by the syntrophic association with bacteria having the conventional WL pathway and/or the alternative GSR pathway, which is mediated by the glycine cleavage system (GCS).^17,18^ In more detail, WL and GSR pathways can coexist in bacteria, where they utilize similar electron carriers with the difference that the GSR pathway bypasses methylene–tetrahydrofolate (THF).^18^ Analysis of KEGG module M00377 (WL pathway) completeness (Figure S3 of the Supporting Information) confirmed the presence of putative syntrophic acetate oxidizing bacteria (SAOB) in the community, such as *Keratinibaculum* sp. ma44 and *Acetomicrobium* sp. ma133.

Henceforth, the subset of MAGs under consideration will exclusively encompass those exhibiting a RA of 3% or higher in at least one generation across both F2 and P2 reactors because a higher coverage ensures reliability of variant calling. The negative trend in RA and the number of species present was confirmed by the decreasing α diversity in Shannon and Chao1 indices (sheet S13 of the Supporting Information). Several MAGs characterized by a high RA in the beginning disappeared at later stages, probably as a result of their inability to tolerate high TAN or salt concentrations. Conversely, *Clostridium cochlearium* ma73, *Keratinibaculum* sp. ma44, and *M. bourgensis* vb3066 all increased in RA with time (Figure S3 of the Supporting Information). A threshold TAN level was probably reached at the 15th generation, because previously abundant species, including *Acetomicrobium* sp. ma133 and Firmicutes sp. ma48, suddenly experienced severe RA reduction.

The VFA trend along generations was parabolic, peaking at the 10th generation. A decrease in VFA concentrations, especially acetic acid, was observed at the end of the incubation. This may be the effect of a later abundance increment in putative SAOB, such as Tepidanaerobacteraceae sp. ma135 (Figures S3 and S4 and sheet S3 of the Supporting Information).^55^ Additionally, bacteria having high completeness in the butyrate- and/or propionate-degrading KEGG modules, such as *Aminobacterium colombiense* co9 and *Tissierella praeacuta* ma27, showed a matching trend. In particular, *A. colombiense* is reported to be a propionate degrader.^55^ Their degradation activity may be reduced by an increase in primary degraders after the acetate decrease. Specifically, acetate accumulation results in that of propionate as well,^56^ as confirmed by the similar trend of their concentrations.

The principal component analysis (PCA) reported in Figure S7 of the Supporting Information reveals a strong correlation of TAN and TKN with later generations, especially the 15th generation. Notably, *Keratinibaculum* sp. ma44 exhibits an evident relationship with those parameters, thus substantiating the role of this bacterium as putative SAOB, which emerges at high ammonia concentrations (Figure S3 of the Supporting Information).

### 3.4 Variant Selection Determined by the Stepwise NH_4_^+^ Increment

**Figure 3:**
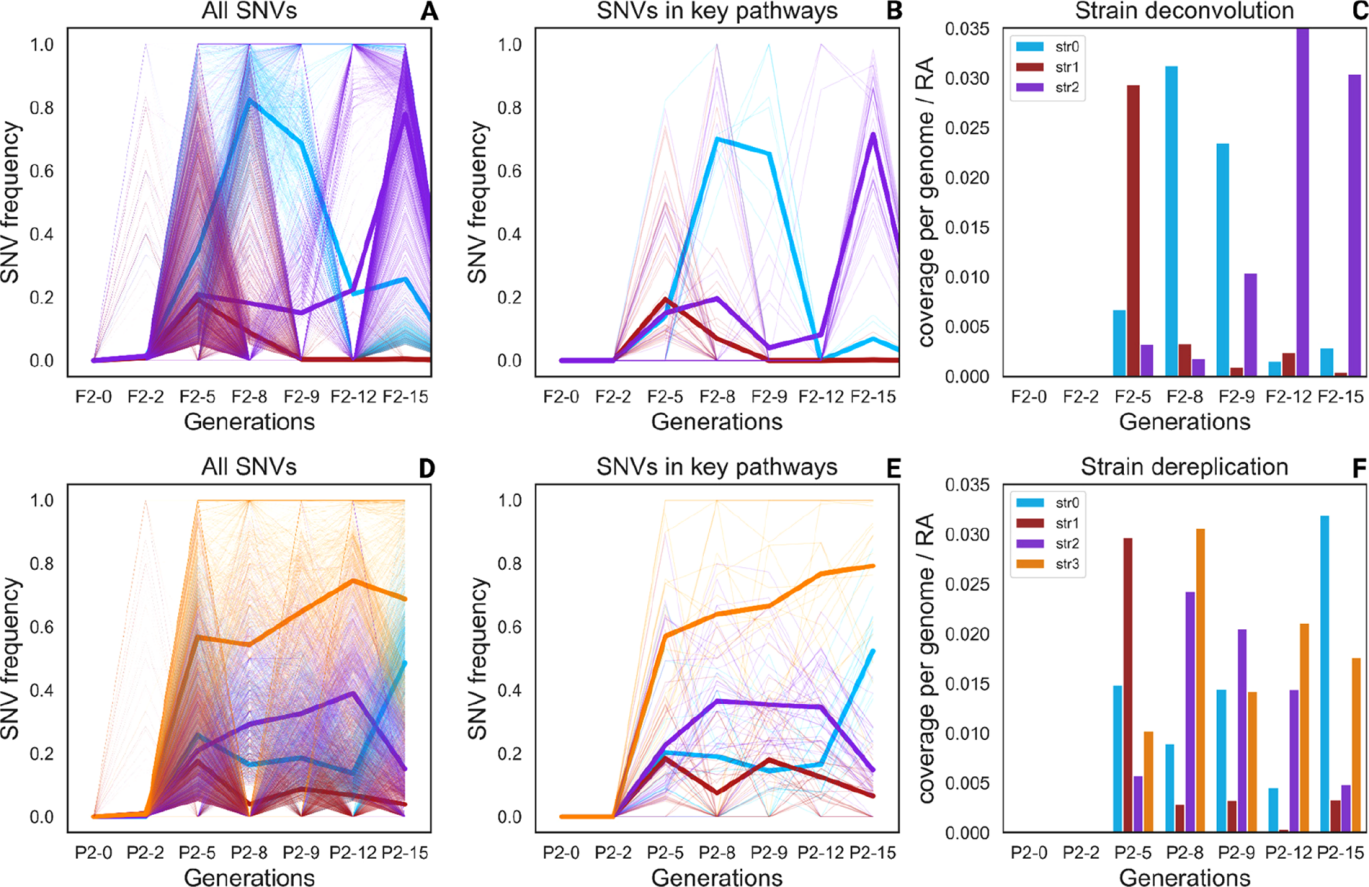
Frequency of nsSNVs over time and strain deconvolution results for *M. bourgensis* vb3066: (A and D) filtered nsSNVs identified in reactors F2 and P2, respectively, (B and E) nsSNVs impacting proteins involved in the hydrogenotrophic metabolism and in the ammonia adaptation process, and (C and F) Strain abundance calculated with STRONG, weighted on the MAG RA. In panels A and D, bold lines represent nsSNV average values for the clusters.

Yenigün and Demirel revealed that changes in TAN and/or FAN can induce deep alterations in the RA of microbial species.^57^ Here, the same effect was also evidenced, and this could be mainly determined by the variable degree of resistance of microbial species to high TAN/FAN. Along with modification in species composition, it must also be considered that microbes can mutate, acquiring an increased resistance to stress.^58^ Indeed, the combination of mutations and the selection of specific mutants determines the development of novel strains according to the environmental pressure. Therefore, in this time-course experiment, nsSNVs were tracked in the 33 most abundant MAGs (Figure 2). Clustering of variants was also implemented to characterize the associated strains over time, thereby assessing their functional response to the NH_4_Cl concentration. Additionally, the trend of SNV frequency was compared to the coverage of strains in the same samples (Figure 3). Again, it must be highlighted that no biological replicates were used in this study, with the limitation that we could not verify if the selective pressure acting on the observed pathways would have equally acted in all of the replicates. The presented results then need to be further validated in future experiments.

Analysis of variants revealed a wide range of genetic heterogeneity among the species. The trend was similar in the two reactors, and the species having a higher number of variants (>2000) at later stages were *Acetomicrobium* sp. ma133, *C. cochlearium* ma73, Firmicutes sp. ma48, Firmicutes sp. mb175, *Keratinibaculum* sp. ma44, *Keratinibaculum* sp. mb43, and *M. bourgensis* vb3066 (Figure S3 of the Supporting Information). To our knowledge, none of these species, except for *M. bourgensis*, were previously reported in the literature as tolerant to a high ammonia concentration. Additionally, resistance mechanisms, such as the metabolic ability to re-equilibrate the cytoplasmic pH, were not previously investigated in these taxa. Considering the microbial ability to persist throughout the experiment, variants present in the genome at later stages are likely to play a role in adaptation.

With regard to the osmotic stress induced by ammonium, a possible resistance mechanism could be related to the regulation of proton balance, potassium uptake, and uptake and/or biosynthesis of osmoprotectants [e.g., glutamate, glutamine, glycine betaine, and *N*^ε^(6)-acetyl-β-l-lysine].^59^ Moreover, because the increased osmotic pressure is highly energy-demanding, microorganisms could be required to boost adenosine 5′-triphosphate (ATP) production. In both bacteria and methanogens, several energy-converting complexes were demonstrated to act toward the replenishment of the H^+^ gradient across the membrane (e.g., Nuo, Hdr, Fwd, Fdh, Mnh, Eha/b, and Ech).^60−62^ Functional analysis of SNVs was employed to investigate the selective pressure on key genes in the subset of seven microbes characterized by over 2000 SNVs in generations 12 and 15, as previously discussed.

#### 3.4.1 Genomic Response and NH_4_^+^ Resistance Mechanism in *M. bourgensis* vb3066

Overall, 12 000 unique SNVs were identified in *M. bourgensis* vb3066 (Figure S3 and section S7 of the Supporting Information). These variants and their change in frequency were linked to the strains and how they evolved along the generations. Consistently, SNVs associated with the same strain tend to have coherent behavior. The SNV trends show that a mixture of strains was already present at the early stages of incubation (Figure 3), and some of them became dominant and exclusive when the TAN concentration became selective. This result is confirmed by strain deconvolution (Figure 3). Analysis of the RA profiles of these strains suggested different levels of resistance. Throughout the experimental period in F2, the dominant strain changed from str1 to str0 and then to str2 in the last two generations. In reactor P2, the dynamics were slightly different, with the dominant strain being str1 at generation five and then shifting to str3 and str0. At generation 15, str2 became dominant but str3 was still present at high abundance. The switch from one strain to another is likely to be determined by their ability to adapt to changing conditions, such as in the case of str2 in F2 and str3 in P2 (Figure 3). To investigate the mechanism triggering a response to stress adaptation, selected variants were linked to the corresponding gene and then prioritized according to the gene function.

**Figure 4:**
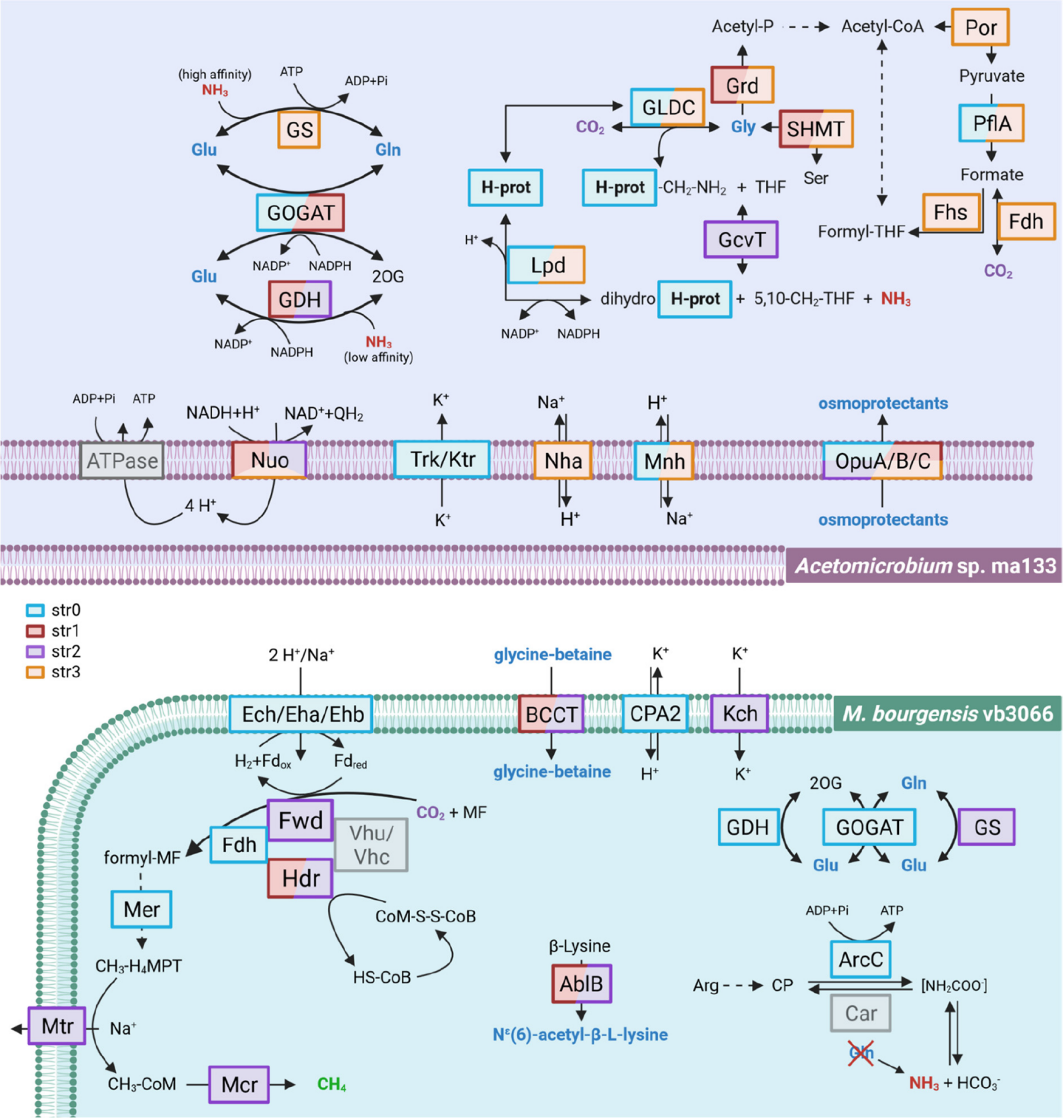
Proposed mechanisms of adaptation to high TAN for *Acetomicrobium* sp. ma133 and *M. bourgensis* vb3066 based on the genes found to accumulate variants by the strain-resolved metagenomic analysis. 2OG, 2-oxoglutarate; H-prot, protein H of GCS (lipoylprotein); THF, tetrahydrofolate; Q, ubiquinone; Fd, ferredoxin; MF, methanofuran; H_4_MPT, tetrahydromethanopterin; CoM, coenzyme M; HS-CoB, coenzyme B; and CoM-S-S-CoB, coenzyme M-HTP heterodisulfide. Osmoprotectants are highlighted in blue. Protein frame colors reflect the strains possessing corresponding SNVs, according to the legend. Mutated enzymes are fully colored, and not mutated enzymes are lightly colored.

An enrichment of nsSNVs on genes associated with the hydrogenotrophic methanogenesis was detected, even though it was not statistically significant (*p* = 0.17 for F2 and *p* = 0.08 for P2). Moreover, during generations five, eight, and nine, a total of 50 variants were found with high frequency on genes associated with potassium uptake, proton balance, synthesis and transport of osmoprotectants, and methanogenesis (panels B and E of Figure 3 and Figure 4 and Table S3 of the Supporting Information). These variants were associated with str0, while those specifically identified at generation F2-12 were associated with str2, which dominated at later stages.

It is known that energy obtained from the substrate-level phosphorylation and multiple energy-converting hydrogenases are crucial in *M. bourgensis* to balance the ATP depletion determined by anabolic activities.^59^ nsSNVs associated with these genes, including *eha*, *ehb*, and *ech*, could be involved in the adaptation of str0 to the increased ammonia concentrations. However, as the community reached generation F2-12, str0 was replaced by str2, which was characterized by 10 denovo nsSNVs associated with genes involved in energy conservation and osmoprotectant synthesis, such as *ablB*, *kch*, and the BCCT family (Table S3 of the Supporting Information). On the other hand, in reactor P2, the strain sweep occurred at generation P2-15, wherein str0 supplanted str3 as predominant. To delve deeper, 11 *de novo* SNVs of str0 were identified within genes associated with glutamate metabolism (*gdh* and *GOGAT*), a recognized osmoprotectant (Figure 4). These results suggest that *M. bourgensis* vb3066 was able to tolerate a strong selective pressure from ammonium. The computed dN/dS ratio confirmed how multiple genes (e.g., *eha*, *glnA*, and *gltD*) were under selection (sheet S12 of the Supporting Information). Furthermore, some of these SNVs scored high on the Grantham distance scale,^49^ particularly in the case of *glnA* and *gltD*, where at least one variant surpassed a value of 160 (sheets S10 and S12 of the Supporting Information). This indicates a substantial amino acid alteration, potentially impacting the structure of the protein. The adaptive capacity may be driven by the selection of strains with superior fitness as a result of beneficial mutations, enabling heightened adaptation to extreme conditions. The enhanced survival rate could be determined by the accumulated mutations linked with genes involved in homeostatic systems, specific energy conservation strategies, and ATP generation via substrate-level phosphorylation and/or driven by an ion gradient.

#### 3.4.2 Enhanced Syntrophic Interactions between Methanogens and SAOB in Response to NH_4_^+^ Stress

Given the rise in RA of *M. bourgensis* vb3066 over the final generations, we hypothesized that it outcompeted the other methanogens by establishing a syntrophic relationship with SAOB. Among the six bacterial MAGs selected for variant analysis, *Acetomicrobium* sp. ma133 was identified as the putative SAOB,^63^ and it was revealed to have over 20 000 unique SNVs (Figure S3 and section S7 of the Supporting Information). Similarly with section 3.4.1 results, SNV clustering revealed the presence of four distantly related strains, a result also confirmed by strain deconvolution (Figure S5 of the Supporting Information). Conversely, no strain replacement events were observed with the increase in the TAN concentration, with one strain being persistently dominant.

To confirm the presence of a mechanism triggering a response to ammonia, variants under positive selection were also specifically investigated in *Acetomicrobium* sp. ma133. A total of 80 nsSNVs with high frequency in the first seven generations were associated with genes coding for enzymes involved in proton balance, potassium uptake, synthesis, and transport of osmoprotectants (Figure 4), as listed in Table S3 of the Supporting Information. The variants on osmoprotectant transporter *opuC*, Na^+^/H^+^ antiporters (*nhaC* and *mnhD*), and glycine-related genes (*glyA* and *grdE*) exhibited a moderate-to-high putative impact in terms of the Grantham distance,^49^ with values of at least 94 for the amino acid substitution (sheets S10 and S12 of the Supporting Information). Moreover, these genes were shown to be under selective pressure, with genes like *grd*, *arcC*, *nha*, and *opuC* having a dN/dS ratio above 1 (sheet S12 of the Supporting Information). In more detail, the dominant strain str3 accumulated nsSNVs primarily in genes related to glycine metabolism, such as *grd* and *GLDC*, along with glutamine (i.e., *glnA*) (Figure 4).

Genes associated with the WL or GSR pathway showed a significant increase in nsSNVs in both reactors, with *p* values of 0.02 (F2) and 0.04 (P2). Furthermore, 148 nsSNVs were linked to genes coding for key enzymes of the WL or GSR pathway in *Acetomicrobium* sp. ma133. This result outnumbers those identified in other bacterial species, supporting the hypothesis of a strong selection acting on SAO. Nevertheless, after the 15th generation, *Acetomicrobium* sp. ma133 underwent a severe drop in RA. As a result of the functional redundancy existing in the AD microbiome, it can be hypothesized that the syntrophic niche was occupied by another organism. The increase in RA (Figure S3 of the Supporting Information) and the presence of several genes related to the WL or GSR pathway (sheet S8 of the Supporting Information) in *C. cochlearium* ma73, *Keratinibaculum* sp. ma44, and Tepidanaerobacteraceae sp. ma135 suggest that either one or all of these bacteria took over as putative SAOB. Overall, these results highlight that the strain evolution driven by increasing ammonium stress is potentially involved in the cooperation between putative SAOB, aiding proper functioning in the experimental conditions. As a result of the stochastic nature of the effect of mutations on microbial genomes, the initially superior fitness of one strain may be surpassed by others acquiring beneficial mutations that confer them a better fitness in a fluctuating dynamic.

### 3.5 Adaptive Strategies and Synergistic Interactions of NH_4_^+^-Tolerant Microbes in AD

Microbial species showed different tolerance levels to a stepwise increase in the TAN concentration. As the TAN level rose, the community underwent a progressive selection and a few highly specialized microbes were able to survive. The presence of multiple strains was defined here, also showing their peculiar trends along the generations and their response to the increased NH_4_Cl level with an unprecedented level of detail. Strain deconvolution revealed an impressive intraspecies diversity, with multiple coexisting strains having different phenotypes as confirmed by their differential response to a high ammonia concentration. It can be speculated that strains represent an additional level of functional redundancy within the microbial community. In general, K^+^ deficiency and the unbalanced osmotic pressure caused by ammonia protonation determined an increased ATP requirement.^59^ To cope with these stressful conditions, fine-tuning of the homeostatic system and deployment of energy conservation strategies are required. The regulation of the turgor pressure and the maintenance of cytoplasmic pH are achieved through systems such as Nha and Trk.^62^ Moreover, the synthesis of compatible solutes enhances survival at a high osmotic pressure.^64^ Multiple energy-converting hydrogenases present supported ion-gradient ATPase functioning through their action in redirecting the electron flux, enabling microorganisms to survive even at high TAN levels (sheet S9 of the Supporting Information). The SNV trend was detected in both *M. bourgensis* vb3066 and *Acetomicrobium* sp. ma133, suggesting that these enzymes are under strong selective pressure (Figure 4). Moreover, most of the genes were selected in strains that, in turn, show a tendency to stabilize and become dominant in the microbiome over time (sheets S8 and S9 of the Supporting Information). This is probably as a result of their key roles in counteracting the inhibition effect and ensuring the survival of microbes by establishing a solid syntrophic relationship.

Altogether, the results obtained in this study provide new insights into AD community evolution and the microbial interactions occurring during adaptation to severely inhibiting conditions. Through metagenomics, the presence of the syntrophies in ammonia-stressed AD reactors was confirmed, but the presence of multiple players working as SAOB and acting at different levels of inhibition was also suggested. Moreover, the findings of this study suggest that there may be a link between the selected accumulated variants on key genes and the capacity of related microbes to survive up to 20 g of NH_4_^+^ N/L. Finally, we were able to demonstrate the feasibility of reaching this TAN level, still with a detectable metabolic activity from the methanogenic community. *M. bourgensis* vb3066 especially withstands this extreme level of TAN, as evidenced by methane production at the end of the observed adaptation period. Overall, our findings highlight the viability of applying strain-resolved metagenomics in a multi-level context, thereby establishing a foundation for further investigations involving more complex substrates. Such outcomes offer potential applications within a bioaugmentation framework, where sharply enriched cultures may reinforce the resident community, thus improving the overall process.

## Supporting Information Available

Raw data and graphs (XLSX)
Figures, tables, and text sections (PDF)
