# Supplementary material for: Adaptation of Anaerobic Digestion Microbial Communities to High Ammonium Levels: Insights from Strain-Resolved Metagenomics
Source: Environ Sci Technol. 2023 Dec 19;58(1):580–90. doi: 10.1021/acs.est.3c07737 (PMC10785762; doi:10.1021/acs.est.3c07737)
Supplement: Supplementary file 2 — es3c07737_si_002.pdf [file es3c07737_si_002.pdf]

Supporting Information

Adaptation of Anaerobic Digestion Microbial Communities to High Ammonium Levels: Insights from Strain-Resolved Metagenomics

Luca Bucci[1](#), Gabriele Ghiotto[1](#), Guido Zampieri[1], Roberto Raga[2], Lorenzo Favaro[3], Laura Treu[1](*), Stefano Campanaro[1]

[1] Department of Biology (DIBIO), University of Padua, via U. Bassi 58/b, 35131 Padova, Italy
[2] Department of Civil, Environmental and Architectural Engineering (ICEA), University of Padua, via Marzolo 9, 35131 Padova, Italy
[3] Department of Agronomy Food Natural resources Animals and Environment (DAFNAE), University of Padua, Agripolis, Viale dell'Università 16, 35020 Legnaro, Italy

(*) Corresponding author: LT (email: laura.treu@unipd.it)
(#) L.B. and G.G. equal contribution.

Summary

Supplementary figures 2
Figure S1: Phylum-level taxonomic classification of F and P inocula 2
Figure S2: Cumulative methane production 3
Figure S3: RA, SNVs and KEGG modules of the most abundant MAGs 4
Figure S4: Single VFA concentrations. 5
Figure S5: Frequency of nsSNVs over time and strain deconvolution for *Acetomicrobium* sp. mA.133 6
Figure S6: Hierarchical clustering of SNVs based on their frequency trends 7
Figure S7: PCA of the biochemical parameters with the RPKM from the reconstructed MAGs 8
Supplementary tables 9
Table S1: Inoculum plan in glucose and acetate 9
Table S2: NH_4Cl, Cl^- and corresponding TAN concentrations 10
Table S3: Genes with detected nsSNVs involved in the proposed mechanisms for ammonia resistance 12
Supplementary text 15
Section S1: Inoculum information 15
Section S2: Gas chromatograph configuration 16
Section S3: DNA sequencing 17
Section S4: Metagenomic data analysis 18
Section S5: Variant- and strain-level analysis 19
Section S6: Glucose- and Acetate-fed reactors performances 20
Section S7: Metagenomic and variants results 21
Section S8: Observation of biofilm formation 22
References 23

Supplementary figures

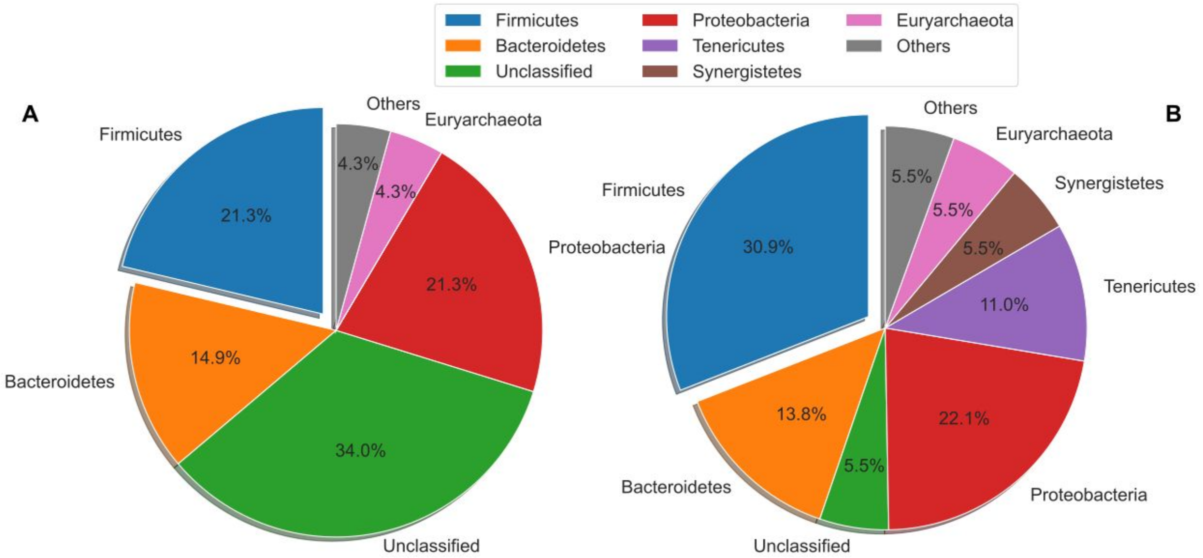

Figure S1: Phylum-level taxonomic classification of F (A) and P (B) inocula. "Others" includes: Spirochaetes, Cloacimonetes, Halanaerobiaeota, Actinobacteria, Patescibacteria, Atribacteria, Thermotogae, Chloroflexi, Verrucomicrobia, Epsilonbacteraeota.

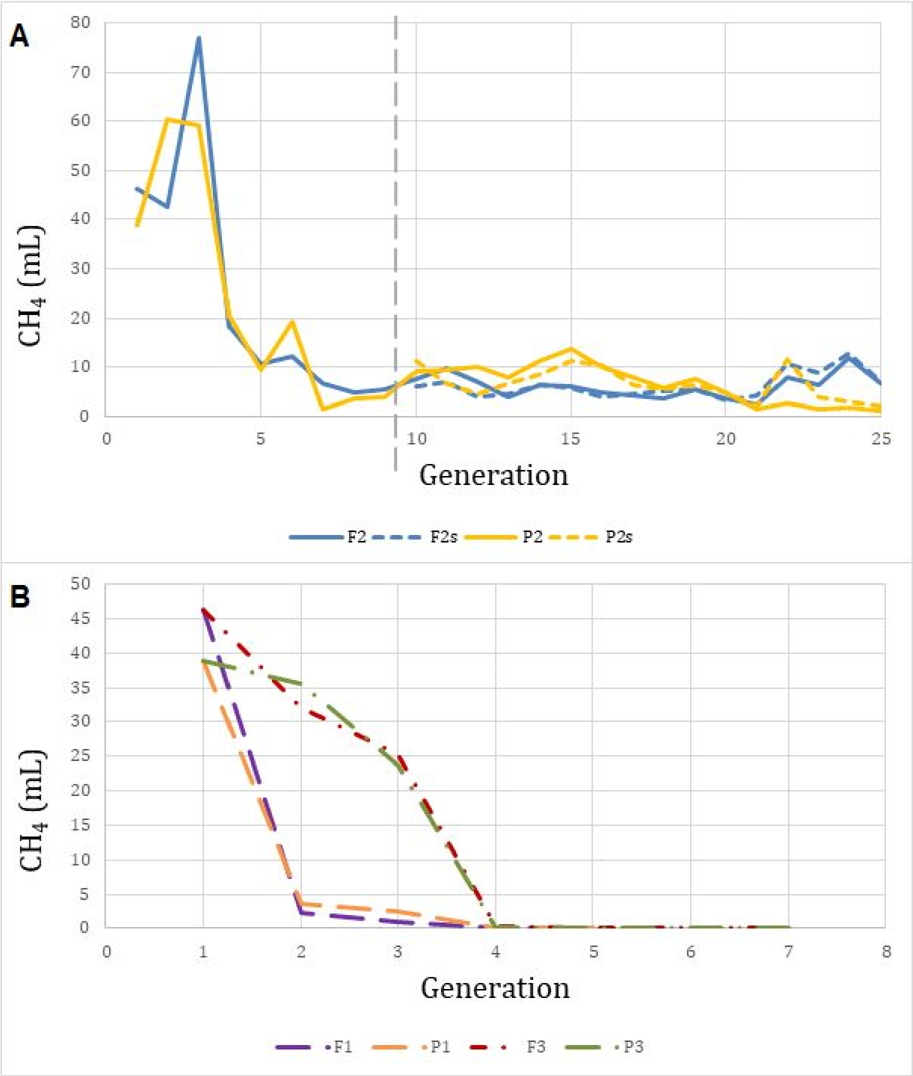

Figure S2: Cumulative methane production for the reactors fed with casein (**A**) and those fed with glucose and acetate (**B**). Numbers after F and P identify the substrate: glucose (1), casein (2), acetate (3).

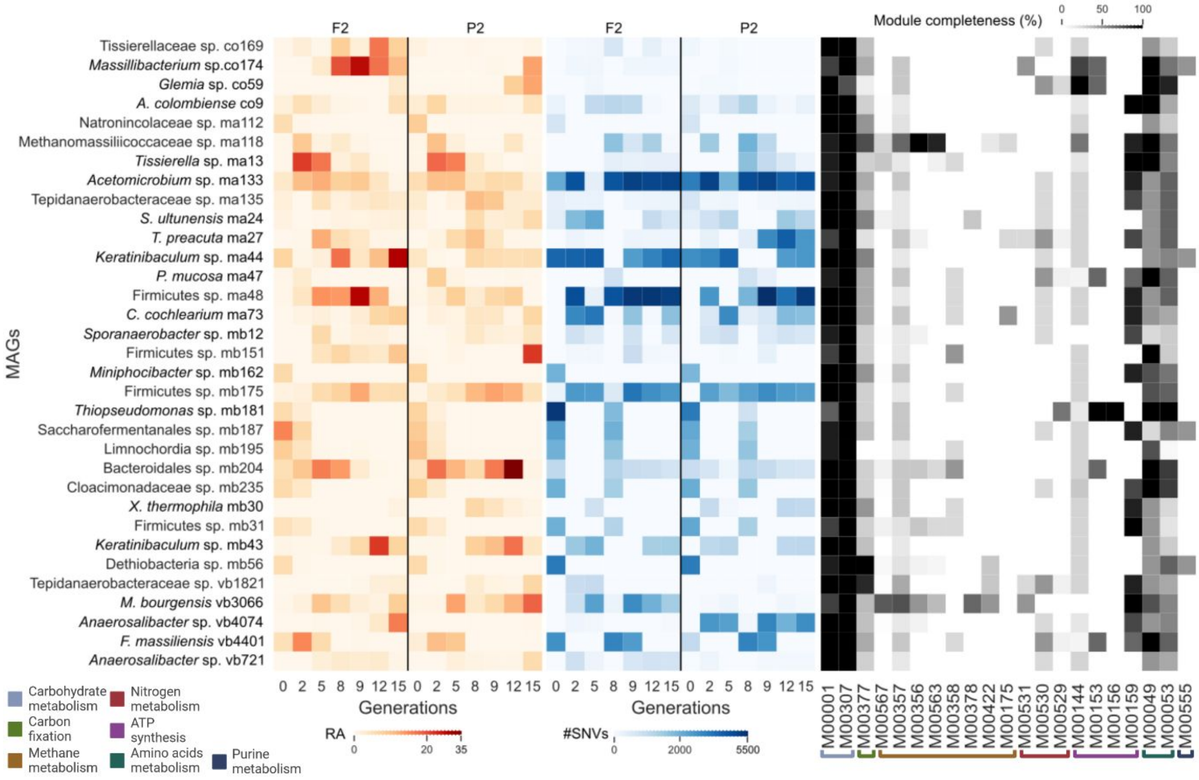

Figure S3: Response of the most abundant MAGs to increasing ammonium concentration. From left to right the heatmaps show RA values (orange), the number of SNVs (blue) and the completeness of relevant KEGG modules (grey). Only the MAGs with RA equal to or higher than 3% in at least one generation are reported.

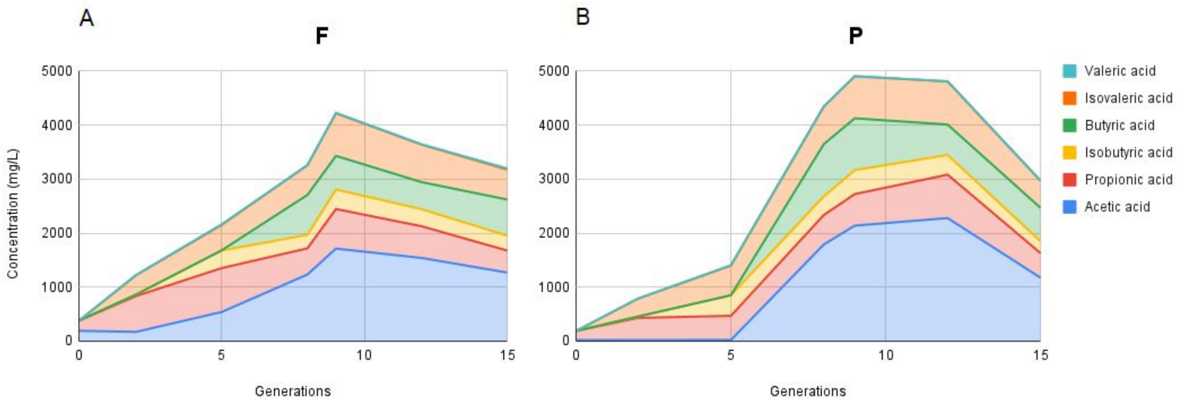

Figure S4: Single VFA concentrations in the selected timepoints.

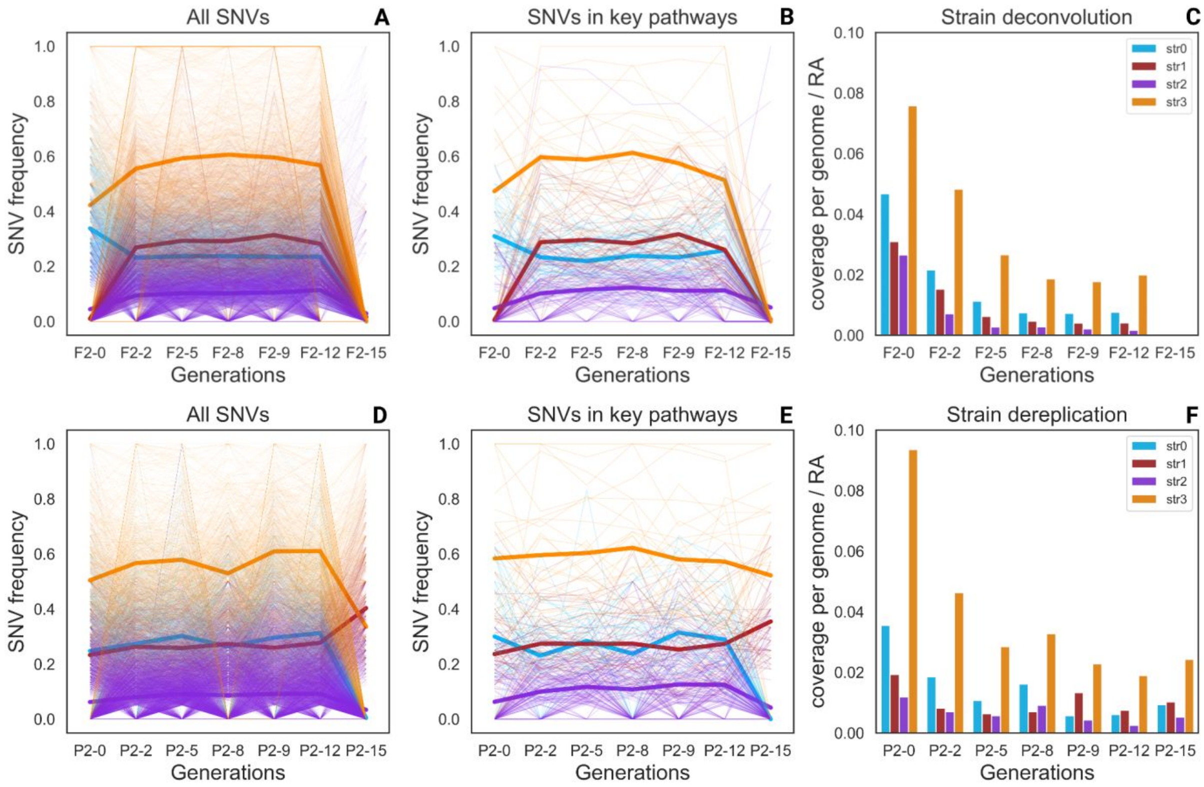

Figure S5: Frequency of nonsynonymous SNVs over time and strain deconvolution results for *Acetomicrobium* sp. mA.133. (A, D) filtered SNVs for reactors F2 and P2, respectively, (B, E) SNVs impacting proteins involved in the ammonia adaptation process, (C, F) abundance of strains calculated by STRONG, weighted on the results of the RA. Bold lines represent SNVs averages.

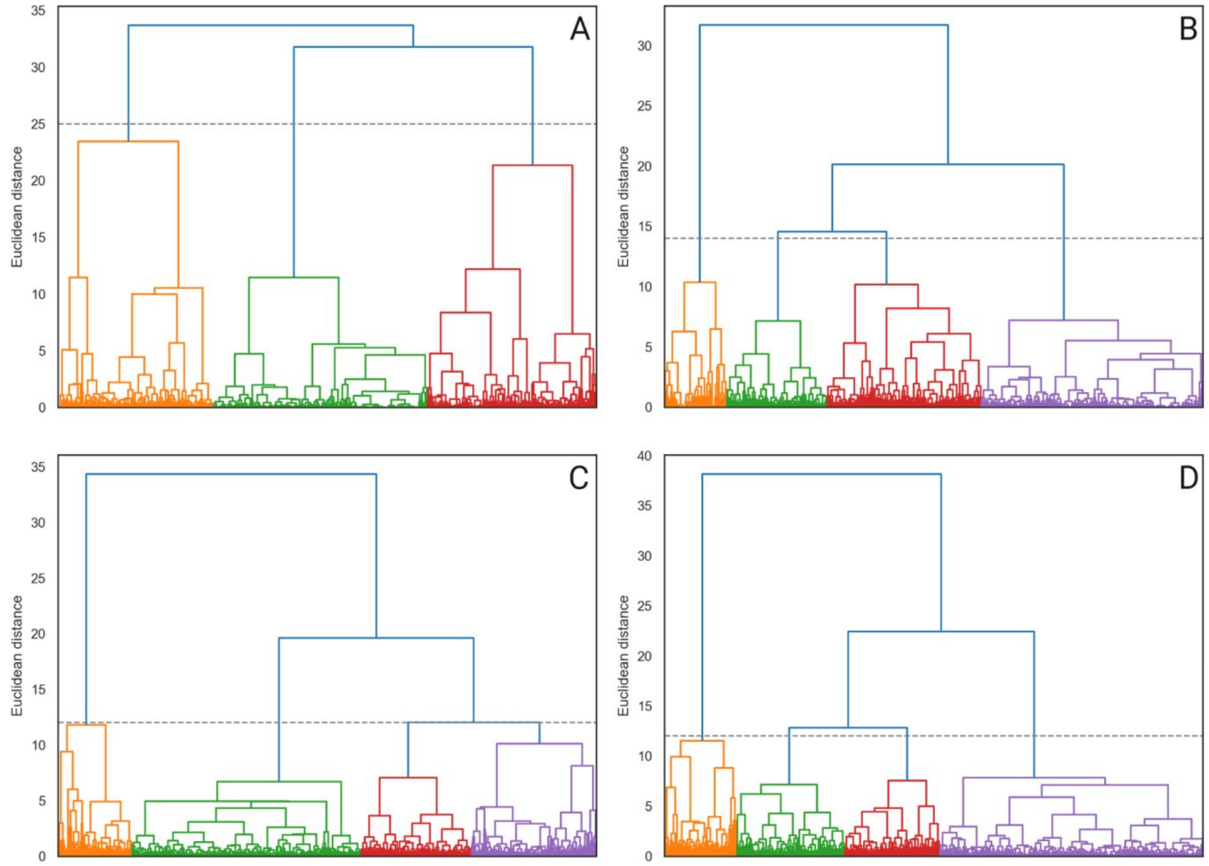

Figure S6: Hierarchical clustering of SNVs based on their frequency trends through time. (A, B) *M. bourgensis* vb3066, respectively in F2 and P2, and (C, D) *Acetomicrobium* sp. ma133, respectively in reactor F2 and P2.

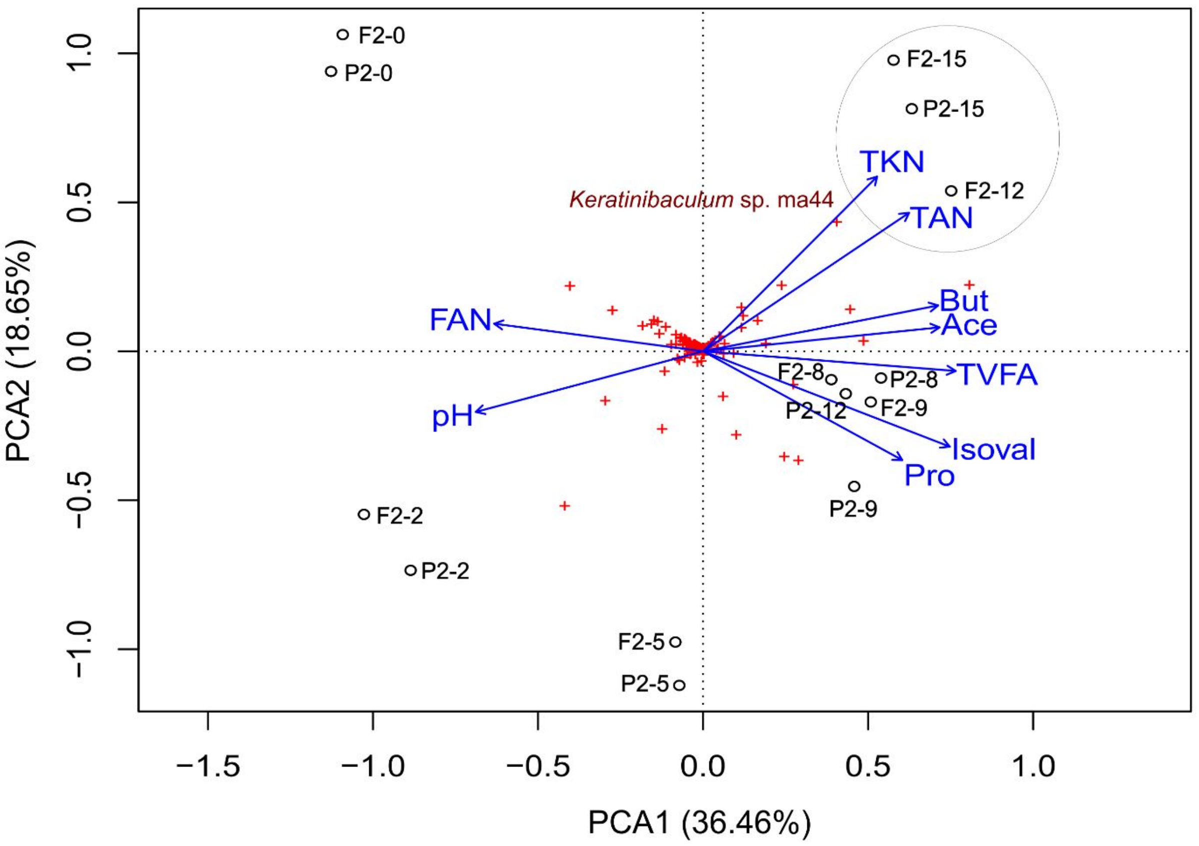

Figure S7: PCA performed by integrating the biochemical parameters measured across generations (pH, TAN, FAN, TKN, VFA) with the RPKM values calculated from the reconstructed MAGs using CoverM v0.6.1. The distance used to plot the PCA was Bray-Curtis.

Supplementary tables

Table S1: Inoculum plan for acclimation procedure in glucose and acetate tests.

	1st Generation	Glucose - Following Generations	Casein – Following Generations	Acetate - Following Generations
**NH_4Cl**	4.5 g/L	+ 1 g/L*	+ 1 g/L*<br>+ 4 g/L†	+ 1 g/L*
**Glucose**		**10 g/L**		
**Casein**			**8 g/L**	
**Acetate**				**2 mL/L**
**YE in BAm (20 g/L)**		10 mL/L	10 mL/L	10 mL/L
**Na_2S (25 g/L)**	10 mL/L	10 mL/L	10 mL/L	10 mL/L
**Vitamin solution (1X)**	20 mL/L	20 mL/L	20 mL/L	20 mL/L

*Increase of NH_4Cl at each re-inoculum (1 g/L corresponds to 247 mg NH_4^+-N/L).

† Increase of NH_4Cl starting from the 11th generation.

Table S2: Concentrations of NH_4Cl and corresponding TAN used for the acclimation process.

	**F/P2**		**F/P2s**			
**Generations**	**NH_4Cl (g/L)**	**Cl^- (g/L)**	**NH_4Cl (g/L)**	**Cl^- (g/L)**	**calculated TAN (mg/L)**	
**1**	4.5	3.0	-	-	**1110**	-
**2**	5.5	3.6	-	-	**1357**	-
**3**	6.5	4.3	-	-	**1603**	-
**4**	7.5	5.0	-	-	**1850**	-
**5**	8.5	5.6	-	-	**2097**	-
**6**	9.5	6.3	-	-	**2343**	-
**7**	10.5	7.0	-	-	**2590**	-
**8**	11.5	7.6	-	-	**2837**	-
**9**	12.5	8.3	-	-	**3083**	-
**10/s***	13.5	8.9	15.5	10.3	**3330**	**3823**
**11/s**	16.5	10.9	18.5	12.3	**4070**	**4563**
**12/s**	20.5	13.6	22.5	14.9	**5056**	**5550**
**13/s**	24.5	16.2	26.5	17.6	**6043**	**6536**
**14/s**	28.5	18.9	30.5	20.2	**7030**	**7523**
**15/s**	32.5	21.5	34.5	22.9	**8016**	**8510**
**16/s**	36.5	24.2	38.5	25.5	**9003**	**9496**
**17/s**	40.5	26.8	42.5	28.2	**9990**	**10483**

**18/s**	44.5	29.5	46.5	30.8	**10976**	**11469**
**19/s**	48.5	32.1	50.5	33.5	**11963**	**12456**
**20/s**	52.5	34.8	54.5	36.1	**12949**	**13443**
**21/s**	56.5	37.4	58.5	38.8	**13936**	**14429**
**22/s**	60.5	40.1	62.5	41.4	**14923**	**15416**
**23/s**	64.5	42.8	66.5	44.1	**15909**	**16403**
**24/s**	68.5	45.4	70.5	46.7	**16896**	**17389**
**25/s**	72.5	48.1	74.5	49.4	**17882**	**18376**
**26/s**	76.5	50.7	78.5	52.0	**18869**	**19362**
**27/s**	80.5	53.4	82.5	54.7	**19856**	**20349**

*Starting from the 10th generation, two different concentrations have been adopted in parallel (distinguished by the presence of an "*s*" after the number).

Table S3: Genes with detected nsSNVs involved in the proposed mechanisms for ammonia resistance in *M. bourgensis* vb3066 and *Acetomicrobium* sp. ma133 and involved in methanogenesis in *M. bourgensis* vb3066.

Category	Protein symbol	Gene name	Function	Microorganism
Transporters	CPA2	Monovalent cation/proton antiporter-2	Re-equilibrium of K^+ levels[1]	*M. bourgensis* vb3066
	Kch	Voltage-gated potassium channels	Re-equilibrium of K^+ levels[2]	*M. bourgensis* vb3066
	Trk	Trk transporter	K^+ uptake[3]	*Acetomicrobium* sp. ma133
	Ktr	Ktr potassium uptake system	Na^+ dependent K^+ uptake[3]	*Acetomicrobium* sp. ma133
	NhaC	Na^+/H^+ antiporter	Na^+ uptake and H^+ efflux. It modestly contributes to pH homeostasis[4]	*Acetomicrobium* sp. ma133
	Mnh	Na^+/H^+ antiporter	Na^+ uptake and H^+ efflux[5]	*Acetomicrobium* sp. ma133
	Nuo	NADH-ubiquinone oxidoreductase	Electrons transfer from NADH to quinone, thus conserving energy by pumping H^+ from the cytoplasm to the periplasm[6]	*Acetomicrobium* sp. ma133
Osmoprotectant transport and synthesis	GS	Glutamine synthase	Synthesis of Gln[7]	*M. bourgensis* vb3066 and *Acetomicrobium* sp. ma133
	GOGAT	Glutamate synthase	Synthesis of Glu[7,8]	*M. bourgensis* vb3066 and *Acetomicrobium* sp. ma133
	GDH	Glutamate dehydrogenase	Synthesis of Glu[8]	*M. bourgensis* vb3066 and *Acetomicrobium* sp. ma133
	BCCT family	Betaine/choline/carnitine transporter	Exogenous glycine betaine import from the environment (which is alternatively produced starting from choline)[9]	*M. bourgensis* vb3066
	AblB	β-lysine N(6)-acetyltransferase	Synthesis of N^{ε}(6)-acetyl-β-L-lysine[10]	*M. bourgensis* vb3066

	Opu	Osmoprotectant uptake systems	Uptake of a broad spectrum of compatible solutes[11]	*Acetomicrobium* sp. ma133
Methanogenesis, hydrogenases	Ech	Ech hydrogenase	Reduction of different ferredoxins, needed in the first step of methanogenesis[12]	*M. bourgensis* vb3066
	Eha-Ehb	Energy-converting hydrogenase	Reduction of different ferredoxins, needed in the first step of methanogenesis[12]	*M. bourgensis* vb3066
	Fdh	Formate dehydrogenase	Reversible formate oxidation[13]	*M. bourgensis* vb3066 and *Acetomicrobium* sp. ma133
	Fwd	Formylmethanofuran dehydrogenase	Reversible formylmethanofuran dehydrogenation[14]	*M. bourgensis* vb3066
	HdrA2	Heterodisulfide reductase	Part of a bifurcating multienzyme complex, involved in energy conservation and responsible for the coupling of ferredoxin reduction[15]	*M. bourgensis* vb3066
	Mer	Methylenetetrahydromethanopterin reductase	Reduction of methylene-H_4MPT to methyl-H_4MPT[16]	*M. bourgensis* vb3066
	Mtr	Tetrahydromethanopterin S-methyltransferase	Energy-conserving sodium-ion translocating pump involved in the methanogenesis[17]	*M. bourgensis* vb3066
	Mcr	Methyl-CoM reductase	Catalyses the last step of the methanogenesis[18]	*M. bourgensis* vb3066
Glycine cleavage system (GCS)	GLDC	Glycine decarboxylase (P Protein)	Decarboxylation of glycine and methylamine transfer to H protein (first step in the GCS)[19]	*Acetomicrobium* sp. ma133
	GcvT	Aminomethyltransferase (T protein)	Aminomethyl transfer to THF (second step in the GCS)[19]	*Acetomicrobium* sp. ma133
	Lpd	Dihydrolipoyl dehydrogenase (L protein)	Electron transfer, with NADH regeneration (third step in the GCS)[19]	*Acetomicrobium* sp. ma133

	GcvH	H Protein	Carrier protein between P, T, and L proteins[19]	*Acetomicrobium* sp. ma133
WL/GSR pathway	Fhs	Formyltetrahydrofolate synthetase	Conversion of formate + THF to formyl-THF[20]	*Acetomicrobium* sp. ma133
	Grd	Glycine reductase	Reduction of Gly to acetyl-CoA[20]	*Acetomicrobium* sp. ma133
	SHMT	Serine hydroxymethyltransferase	Reversible conversion of Gly to Ser[20]	*Acetomicrobium* sp. ma133
	Por	Pyruvate synthase	Reversible conversion of pyruvate to acetyl-CoA[20]	*Acetomicrobium* sp. ma133
				Acetomicrobium sp. ma133
Others	Pfl	Pyruvate-formate lyase	Reversible conversion of pyruvate to formate[21]	*Acetomicrobium* sp. ma133
	ArcC	Carbamate kinase	Carbamoyl phosphate to carbamate conversion, in the Arg deiminase pathway adopted for anaerobic energy generation. In its reverse synthesis path, NH_3 at high concentrations replaces Gln as N donor[22]	*M. bourgensis* vb3066

Supplementary text

Section S1: Inoculum information

The mesophilic full-scale AD plant chosen for the sampling is constituted by three subsequent tanks, each dedicated to a different step, namely hydrolysis, fermentation, and post-fermentation. At the time of sampling, the biogas plant was fed daily with approximately 30 tons of poultry manure, 23.5 tons of maize silage, 2 tons of maize flour, 25 m^3 of swine sewage, 25 m^3 of cattle sewage, and 30 m^3 of water.

Section S2: Gas chromatograph configuration

The gas was analysed using a gas chromatograph (8860 GC, Agilent Technologies, Santa Clara, CA, USA) equipped with a thermal conductivity detector (TCD). Three micro packed columns were used for the gas separation, namely Hayesep Q (1.5 m, 1/16" OD, 1.0 mm ID), Hayesep N (0.5 m, 1/16" OD, 1 mm ID) and MolSieve 5Å (1.5 m, 1/16" OD, 1.0 mm ID), all using helium as carrier gas. Volatile fatty acids (VFA) concentrations were determined from the liquid phase using the same 8860 GC (Agilent) equipped with a flame ionisation detector (FID) and a DB-FFAP fused silica capillary column (30 m, 0.25 mm ID, film thickness 0.25 µm) using helium as carrier gas. All chromatograms were analysed through the ChemStation software (B.04.03-SP2, Agilent).

Section S3: DNA sequencing

The Nanopore library was prepared using the SQK-RBK004 rapid barcoding kit (Oxford Nanopore Technologies, UK), according to the manufacturer's instructions. Then, the library was loaded onto a FLO-MIN106 flow cell, and sequenced with a MinION platform (Oxford Nanopore Technologies). The sequencing run was monitored using MinKNOW v21.06.0 software. Guppy v5.0.11 base-calling tool was used for translating raw electrical signals to nucleotide sequences.

Section S4: Metagenomic data analysis

The metagenomic pipeline employed is reported in the Materials and Methods section 2.5. CoverM v0.6.1[23] was used to calculate the read count and the RPKM on the reconstructed MAGs. The alpha-diversity was calculated with the Phyloseq v1.40.0 package[24] embedded in R v4.3.1, using as input the previously calculated mapped read count. Shannon's and Chao1's indexes were chosen to represent the result (**SI2 sheet S13**). The PCA was performed using the Vegan v2.6-4 package[25] embedded in R v4.3.1, and the input data were the RPKM calculated on MAGs and the environmental parameters measured (pH, TAN, FAN, TKN, TVFA, Acetate, Butyrate, Propionate, Isovalerate). The function rankindex() was applied to rank Spearman correlations between dissimilarity indices and gradient separation. The index with the higher correlation was Bray-Curtis, which was chosen to perform Distance-based redundancy analysis (dbRDA) using the package capscale(). The resulting biplot was visualised with ggplot2 (**Figure S7**).

Section S5: Variant- and strain-level analysis

The InStrain profile module takes as input a FASTA file containing the whole set of MAGs and a BAM file for each sample obtained from the alignment of the Illumina reads on such MAGs. A scaffold-to-bin file and the gene annotation files were also included in order to produce genome-level and scaffold-level statistics for the variants. InStrain was run using default parameters except for two optional settings: --min_mapq 2 and --min_read_ani 0.98. For downstream analysis, manipulation of these tabular files was performed in Python using numpy (v1.23.5), pandas (v1.5.2), matplotlib (v3.6.2), seaborn (v0.12.1), and scipy (v1.9.3).

The variants clustering approach was implemented using the hierarchical clustering algorithm from the scipy.stats library in Python. The Ward distance metric was used to calculate the similarity between the variants. Moreover, the Mann-Whitney U test was applied to assess the accumulation of nonsynonymous SNVs (nsSNVs) in the genes associated with the pathways of interest in a MAG with respect to the overall gene population harbouring mutations in the same MAG. Specifically, the global distribution of nsSNVs in each gene, normalised by the gene length, was compared with such distribution in the selected pathways. This analysis was conducted using the scipy.stats library in Python, and the "exact" method was employed to calculate the p-value. By comparing the observed statistic against the exact distribution of the statistic under the null hypothesis, this method accurately computes the precise p-value.

The results obtained from the variant calling using InStrain were subjected to manual post-processing to eliminate low-confidence outcomes. First, SNVs situated within a 150 bp proximity to both the 3' and 5'-ends of each scaffold were excluded. This was necessary due to the tendency for coverage to decrease in these genomic regions, potentially compromising result reliability. Next, any variant in which the difference between the SNV position coverage and the average coverage of the scaffold fell outside the interval [-100;+100] was eliminated. Lastly, the ratio between the number of reads supporting the variant allele and those supporting the reference allele was calculated. Any SNVs with a ratio lower than 0.15 in all timepoints were subsequently discarded. Altogether, these steps were crucial for maintaining data reliability, reducing the number of false positives in spite of a loss of true negatives. The procedure allowed the removal of around 20-25% of SNV, depending on the reactor.

Section S6: Glucose- and Acetate-fed reactors performances

In the case of glucose as the main C source, a drop to zero in CH_4 production suggested a system failure. Starting from the 4th generation, the acidification of the medium occurred, coherently with the absence of methane production. Frequently, a drop in pH is associated with an accumulation of short-chain fatty acids determined by a faster metabolic activity of the hydrolytic over acetoclastic species (the primary degraders), in comparison to the transformation rate accomplished by the acetoclastic species. As a consequence, there is a lack of molecules for methanogenesis, resulting in AD failure. For this reason, glucose batches were halted at the 4th generation. In the acetate-fed cultures, the pH remained stable, but a system failure occurred in the 6th and 7th generations, where no methane production was detected.
Different explanations for the selection of low ammonia-resistant species in glucose and acetate-fed batches can be formulated. First, the drastic change in substrate composition could have led to a major loss of protein-degrading species, while the surviving microbes were not able to cope with the increased ammonia concentration. Second, the delicate acetoclastic archaea were the most likely to be negatively selected aiding the system failure. Interestingly, in casein-fed communities, a high and protracted resilience was observed. Noteworthy, methanation occurred still in the last reached generation (27th), although with a very limited cumulative production of methane observed.

Section S7: Metagenomic and variants results

Long and short reads were combined to obtain a high-quality hybrid assembly, for a total length of 618.24 Mb. The average alignment rate of short reads on the assembly was 96.78%, confirming that the microbial community is almost entirely represented. After the binning, 70.69% of the reads were aligned to the MAGs, with values ranging from 56.84 to 80.44% according to the sample. This indicates that the following taxonomic and functional analyses performed on MAGs accurately represent the majority of the microbiome. The integration of multiple binning approaches allowed the identification of 179 MAGs, 99 of them of high quality and 80 of medium quality, according to the minimum information about metagenome-assembled genomes (MIMAG)[26]. A global analysis previously performed on AD systems revealed an average assembly size of 409 Mbp (minimum 47 Mbp, maximum 2.3 Gbp) and an average number of MAGs of 148 (minimum 20, maximum 636 MAGs) for the AD microbiome[27]. This suggests that the community of the current study has an intermediate level of complexity. The taxonomic assignment revealed 7 archaeal (3.91%) and 172 bacterial (96.09%) MAGs. At the Phylum level, the microbiome spans 15 phyla with a striking majority of species assigned to Firmicutes (72.07%). The selecting pressure of stepwise increased ammonia levels shaped the microbial composition inducing a shift towards more resistant and specialised species. Among the methanogens, *Methanoculleus bourgensis* vb3066 appeared to play a dominant role (mean relative abundance (RA) = 4.33%), whereas the other six archaeal species have overall an average RA of 1.88% (**Figure 2**).

SNVs in *M. bourgensis* vb3066 were mostly classified as synonymous (61.22%), while the remaining were nonsynonymous (27.98%), intergenic (10.45%), and multigenic (0.35%). The nsSNVs-impacted genes involved in hydrogenotrophic methanogenesis and mechanisms counteracting ammonia inhibition had a total length of 36246 (in F2) and 64113 (in P2) bp, and represented 2.01 and 3.55% of the genome in reactors F2 and P2, respectively (**SI**). These values are consistent with the analysis performed on the AD database[27], where they don't exceed 10%.

SNVs in *Acetomicrobium* sp. ma133 were annotated as synonymous (61.28%), nonsynonymous (28.14%), intergenic (10.28%), and multigenic (0.30%). The percentage of the genome impacted by nsSNVs linked to genes involved in the WL or GSR pathways, as well as those involved in mechanisms counteracting ammonia inhibition, was 3.09 and 3.14% for reactors F2 and P2, respectively.

Section S8: Observation of biofilm formation

Microbes inhabiting physiologically-unfavourable environments can form biofilms, establishing less stressing environmental niches, in which communities continuously thrive and evolve together[28]. The biofilm serves as a protective and homeostatic stronghold for resident microorganisms, allowing them to adapt to harsh environmental fluctuations[28]. Starting from the 19th generation, biofilm-like structures in the form of pellicles or slimes were evidenced in the batch cultures for the first time (**Figure S8**). This formation was observed in all the following generations. It can be speculated that the biofilm presence favoured the survival of microorganisms in such stressing conditions.

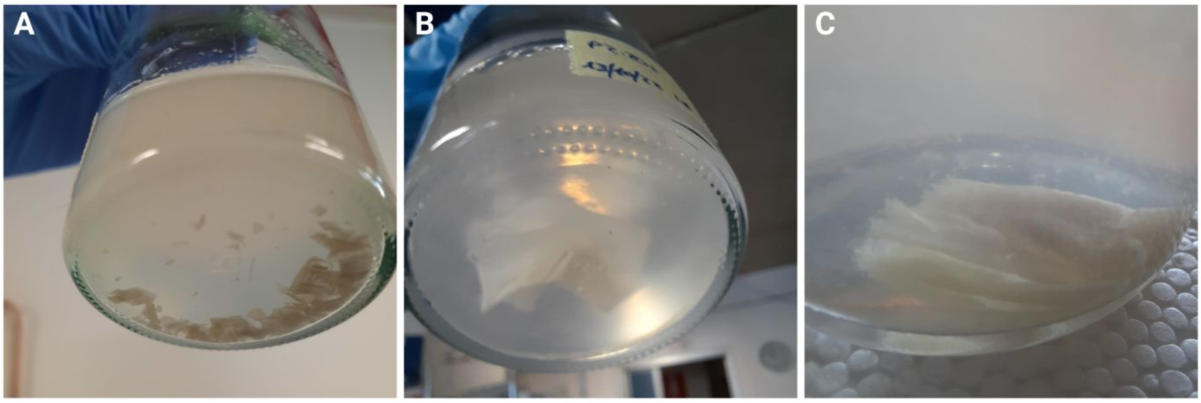

Figure S8: Observed biofilms in the batches P2.19 (**A**), P2.20s (**B**), and F2.27 (**C**).

References

(1) Masrati, G.; Dwivedi, M.; Rimon, A.; Gluck-Margolin, Y.; Kessel, A.; Ashkenazy, H.; Mayrose, I.; Padan, E.; Ben-Tal, N. Broad Phylogenetic Analysis of Cation/Proton Antiporters Reveals Transport Determinants. *Nat Commun* **2018**, *9* (1), 4205. https://doi.org/10.1038/s41467-018-06770-5.

(2) Loukin, S. H.; Kuo, M. M.-C.; Zhou, X.-L.; Haynes, W. J.; Kung, C.; Saimi, Y. Microbial K+ Channels. *J Gen Physiol* **2005**, *125* (6), 521–527. https://doi.org/10.1085/jgp.200509261.

(3) Kraegeloh, A.; Amendt, B.; Kunte, H. J. Potassium Transport in a Halophilic Member of the Bacteria Domain: Identification and Characterization of the K+ Uptake Systems TrkH and TrkI from Halomonas Elongata DSM 2581T. *J Bacteriol* **2005**, *187* (3), 1036–1043. https://doi.org/10.1128/JB.187.3.1036-1043.2005.

(4) Prágai, Z.; Eschevins, C.; Bron, S.; Harwood, C. R. Bacillus Subtilis NhaC, an Na+/H+ Antiporter, Influences Expression of thephoPR Operon and Production of Alkaline Phosphatases. *Journal of Bacteriology* **2001**, *183* (8), 2505–2515. https://doi.org/10.1128/JB.183.8.2505-2515.2001.

(5) Swartz, T. H.; Ito, M.; Ohira, T.; Natsui, S.; Hicks, D. B.; Krulwich, T. A. Catalytic Properties of Staphylococcus Aureus and Bacillus Members of the Secondary Cation/Proton Antiporter-3 (Mrp) Family Are Revealed by an Optimized Assay in an Escherichia Coli Host. *Journal of Bacteriology* **2007**, *189* (8), 3081–3090. https://doi.org/10.1128/JB.00021-07.

(6) Ito, T.; Gallegos, R.; Matano, L. M.; Butler, N. L.; Hantman, N.; Kaili, M.; Coyne, M. J.; Comstock, L. E.; Malamy, M. H.; Barquera, B. Genetic and Biochemical Analysis of Anaerobic Respiration in Bacteroides Fragilis and Its Importance In Vivo. *mBio* **2020**, *11* (1), e03238-19. https://doi.org/10.1128/mBio.03238-19.

(7) Reitzer, L. J.; Magasanik, B. Expression of glnA in Escherichia Coli Is Regulated at Tandem Promoters. *Proc. Natl. Acad. Sci. U.S.A.* **1985**, *82* (7), 1979–1983. https://doi.org/10.1073/pnas.82.7.1979.

(8) Yan, D.; Ikeda, T. P.; Shauger, A. E.; Kustu, S. Glutamate Is Required to Maintain the Steady-State Potassium Pool in Salmonella Typhimurium. *Proc Natl Acad Sci U S A* **1996**, *93* (13), 6527–6531.

(9) Yang, N.; Ding, R.; Liu, J. Synthesizing Glycine Betaine via Choline Oxidation Pathway as an Osmoprotectant Strategy in Haloferacales. *Gene* **2022**, *847*, 146886. https://doi.org/10.1016/j.gene.2022.146886.

(10) Pflüger, K.; Baumann, S.; Gottschalk, G.; Lin, W.; Santos, H.; Müller, V. Lysine-2,3-Aminomutase and β-Lysine Acetyltransferase Genes of Methanogenic Archaea Are Salt Induced and Are Essential for the Biosynthesis of N^{ε}-Acetyl-β-Lysine and Growth at High Salinity. *Appl Environ Microbiol* **2003**, *69* (10), 6047–6055. https://doi.org/10.1128/AEM.69.10.6047-6055.2003.

(11) Du, Y.; Shi, W.-W.; He, Y.-X.; Yang, Y.-H.; Zhou, C.-Z.; Chen, Y. Structures of the Substrate-Binding Protein Provide Insights into the Multiple Compatible Solute Binding Specificities of the Bacillus Subtilis ABC Transporter OpuC. *Biochemical Journal* **2011**, *436* (2), 283–289. https://doi.org/10.1042/BJ20102097.

(12) Anderson, I.; Ulrich, L. E.; Lupa, B.; Susanti, D.; Porat, I.; Hooper, S. D.; Lykidis, A.; Sieprawska-Lupa, M.; Dharmarajan, L.; Goltsman, E.; Lapidus, A.; Saunders, E.; Han, C.; Land, M.; Lucas, S.; Mukhopadhyay, B.; Whitman, W. B.; Woese, C.; Bristow, J.; Kyrpides, N. Genomic Characterization of Methanomicrobiales Reveals Three Classes of Methanogens. *PLOS ONE* **2009**, *4* (6), e5797. https://doi.org/10.1371/journal.pone.0005797.

(13) Yang, J.; Lee, S. H.; Ryu, J.-Y.; Lee, H. S.; Kang, S. G. A Novel NADP-Dependent Formate Dehydrogenase From the Hyperthermophilic Archaeon Thermococcus Onnurineus NA1. *Frontiers in Microbiology* **2022**, *13*.

(14) Bertram, P. A.; Karrasch, M.; Schmitz, R. A.; Böcher, R.; Albracht, S. P. J.; Thauer, R. K. Formylmethanofuran Dehydrogenases from Methanogenic Archaea Substrate Specificity, EPR Properties and Reversible Inactivation by Cyanide of the Molybdenum or Tungsten Iron-Sulfur Proteins. *European Journal of Biochemistry* **1994**, *220* (2), 477–484. https://doi.org/10.1111/j.1432-1033.1994.tb18646.x.

(15) Buckel, W.; Thauer, R. K. Energy Conservation via Electron Bifurcating Ferredoxin Reduction and Proton/Na+ Translocating Ferredoxin Oxidation. *Biochimica et Biophysica Acta (BBA) - Bioenergetics* **2013**, *1827* (2), 94–113. https://doi.org/10.1016/j.bbabio.2012.07.002.

(16) Shima, S.; Warkentin, E.; Grabarse, W.; Sordel, M.; Wicke, M.; Thauer, R. K.; Ermler, U. Structure of Coenzyme F420 Dependent Methylenetetrahydromethanopterin Reductase from Two Methanogenic Archaea. *Journal of Molecular Biology* **2000**, *300* (4), 935–950. https://doi.org/10.1006/jmbi.2000.3909.

(17) Wintsche, B.; Jehmlich, N.; Popp, D.; Harms, H.; Kleinsteuber, S. Metabolic Adaptation of Methanogens in Anaerobic Digesters Upon Trace Element Limitation. *Frontiers in Microbiology* **2018**, *9*.

(18) Bokranz, M.; Bäumner, G.; Allmansberger, R.; Ankel-Fuchs, D.; Klein, A. Cloning and Characterization of the Methyl Coenzyme M Reductase Genes from Methanobacterium Thermoautotrophicum. *Journal of Bacteriology* **1988**, *170* (2), 568–577. https://doi.org/10.1128/jb.170.2.568-577.1988.

(19) Ren, J.; Wang, W.; Nie, J.; Yuan, W.; Zeng, A.-P. Understanding and Engineering Glycine Cleavage System and Related Metabolic Pathways for C1-Based Biosynthesis. In *One-Carbon Feedstocks for Sustainable Bioproduction*; Zeng, A.-P., Claassens, N. J., Eds.; Advances in Biochemical Engineering/Biotechnology; Springer International Publishing: Cham, 2022; pp 273–298. https://doi.org/10.1007/10_2021_186.

(20) Song, Y.; Lee, J. S.; Shin, J.; Lee, G. M.; Jin, S.; Kang, S.; Lee, J.-K.; Kim, D. R.; Lee, E. Y.; Kim, S. C.; Cho, S.; Kim, D.; Cho, B.-K. Functional Cooperation of the Glycine Synthase-Reductase and Wood–Ljungdahl Pathways for Autotrophic Growth of *Clostridium Drakei*. *Proc. Natl. Acad. Sci. U.S.A.* **2020**, *117* (13), 7516–7523. https://doi.org/10.1073/pnas.1912289117.

(21) Stairs, C. W.; Roger, A. J.; Hampl, V. Eukaryotic Pyruvate Formate Lyase and Its Activating Enzyme Were Acquired Laterally from a Firmicute. *Molecular Biology and Evolution* **2011**, *28* (7), 2087–2099. https://doi.org/10.1093/molbev/msr032.

(22) Baur, H.; Luethi, E.; Stalon, V.; Mercenier, A.; Haas, D. Sequence Analysis and Expression of the Arginine-Deiminase and Carbamate-Kinase Genes of Pseudomonas

Aeruginosa. *European Journal of Biochemistry* **1989**, *179* (1), 53–60. https://doi.org/10.1111/j.1432-1033.1989.tb14520.x.
(23) Woodcroft, B. J. CoverM, 2023. https://github.com/wwood/CoverM (accessed 2023-09-15).
(24) McMurdie, P. J.; Holmes, S. Phyloseq: An R Package for Reproducible Interactive Analysis and Graphics of Microbiome Census Data. *PLOS ONE* **2013**, *8* (4), e61217. https://doi.org/10.1371/journal.pone.0061217.
(25) Vegan: An R Package for Community Ecologists, 2023. https://github.com/vegandevs/vegan (accessed 2023-09-15).
(26) Bowers, R. M.; Kyrpides, N. C.; Stepanauskas, R.; Harmon-Smith, M.; Doud, D.; Reddy, T. B. K.; Schulz, F.; Jarett, J.; Rivers, A. R.; Eloe-Fadrosh, E. A.; Tringe, S. G.; Ivanova, N. N.; Copeland, A.; Clum, A.; Becraft, E. D.; Malmstrom, R. R.; Birren, B.; Podar, M.; Bork, P.; Weinstock, G. M.; Garrity, G. M.; Dodsworth, J. A.; Yooseph, S.; Sutton, G.; Glöckner, F. O.; Gilbert, J. A.; Nelson, W. C.; Hallam, S. J.; Jungbluth, S. P.; Ettema, T. J. G.; Tighe, S.; Konstantinidis, K. T.; Liu, W.-T.; Baker, B. J.; Rattei, T.; Eisen, J. A.; Hedlund, B.; McMahon, K. D.; Fierer, N.; Knight, R.; Finn, R.; Cochrane, G.; Karsch-Mizrachi, I.; Tyson, G. W.; Rinke, C.; Lapidus, A.; Meyer, F.; Yilmaz, P.; Parks, D. H.; Murat Eren, A.; Schriml, L.; Banfield, J. F.; Hugenholtz, P.; Woyke, T. Minimum Information about a Single Amplified Genome (MISAG) and a Metagenome-Assembled Genome (MIMAG) of Bacteria and Archaea. *Nat Biotechnol* **2017**, *35* (8), 725–731. https://doi.org/10.1038/nbt.3893.
(27) Campanaro, S.; Treu, L.; Rodriguez-R, L. M.; Kovalovszki, A.; Ziels, R. M.; Maus, I.; Zhu, X.; Kougias, P. G.; Basile, A.; Luo, G.; Schlüter, A.; Konstantinidis, K. T.; Angelidaki, I. New Insights from the Biogas Microbiome by Comprehensive Genome-Resolved Metagenomics of Nearly 1600 Species Originating from Multiple Anaerobic Digesters. *Biotechnology for Biofuels* **2020**, *13* (1), 25. https://doi.org/10.1186/s13068-020-01679-y.
(28) Parrilli, E.; Tutino, M. L.; Marino, G. Biofilm as an Adaptation Strategy to Extreme Conditions. *Rend. Fis. Acc. Lincei* **2022**, *33* (3), 527–536. https://doi.org/10.1007/s12210-022-01083-8.
